# A blood DNA methylation biomarker for predicting short-term risk of cardiovascular events

**DOI:** 10.1186/s13148-022-01341-4

**Published:** 2022-09-29

**Authors:** Andrea Cappozzo, Cathal McCrory, Oliver Robinson, Anna Freni Sterrantino, Carlotta Sacerdote, Vittorio Krogh, Salvatore Panico, Rosario Tumino, Licia Iacoviello, Fulvio Ricceri, Sabina Sieri, Paolo Chiodini, Gareth J. McKay, Amy Jayne McKnight, Frank Kee, Ian S. Young, Bernadette McGuinness, Eileen M. Crimmins, Thalida Em Arpawong, Rose Anne Kenny, Aisling O’Halloran, Silvia Polidoro, Giuliana Solinas, Paolo Vineis, Francesca Ieva, Giovanni Fiorito

**Affiliations:** 1 MOX - Laboratory for Modeling and Scientific Computing, Department of Mathematics, Politecnico di Milano, Milan, Italy; 2 Department of Medical Gerontology, Trinity College Dublin, Dublin, Ireland; 3 MRC-PHE Centre for Environment and Health, Imperial College London, London, UK; 4 The Alan Turing Institute, London, UK; 5 Unit of Cancer Epidemiology, Città della Salute e della Scienza University-Hospital, Turin, Italy; 6 Fondazione IRCCS - Istituto Nazionale dei Tumori, Milan, Italy; 7 Department of Clinical Medicine and Surgery, University of Naples Federico II, Naples, Italy; 8 Association for Epidemiology Research, AIRE ONLYS, Ragusa, Italy; 9 Department of Epidemiology and Prevention, IRCCS NEUROMED, Pozzilli, Italy; 10 Department of Medicine and Surgery, Research Center in Epidemiology and Preventive Medicine (EPIMED), Turin, Italy; 11 Epidemiology Unit, Regional Health Service TO3, Grugliasco, Italy; 12 Department of Clinical and Biological Sciences, Centre for Biostatistics, Epidemiology, and Public Health (C-BEPH), University of Turin, Turin, Italy; 13 Department of Mental, Physical Health and Preventive Medicine, University of Campania ‘Luigi Vanvitelli’, Caserta, Italy; 14 Centre for Public Health, Queen’s University Belfast, Belfast, UK; 15 Leonard Davis School of Gerontology, University of Southern California, Los Angeles, CA USA; 16 Italian Institute for Genomic Medicine (IIGM), Turin, Italy; 17 Laboratory Biostatistics, Department of Biomedical Sciences, University of Sassari, Via Padre Manzella 4, Sassari, Italy; 18 CHDS - Health Data Science Center, Human Technopole, Milan, Italy

**Keywords:** DNA methylation, Molecular epidemiology, Risk scores, Surrogate biomarkers, Cardiovascular risk, Epigenetics

## Abstract

**Background:**

Recent evidence highlights the epidemiological value of blood DNA methylation (DNAm) as surrogate biomarker for exposure to risk factors for non-communicable diseases (NCD). DNAm surrogate of exposures predicts diseases and longevity better than self-reported or measured exposures in many cases. Consequently, disease prediction models based on blood DNAm surrogates may outperform current state-of-the-art prediction models. This study aims to develop novel DNAm surrogates for cardiovascular diseases (CVD) risk factors and develop a composite biomarker predictive of CVD risk. We compared the prediction performance of our newly developed risk score with the state-of-the-art DNAm risk scores for cardiovascular diseases, the ‘next-generation’ epigenetic clock DNAmGrimAge, and the prediction model based on traditional risk factors SCORE2.

**Results:**

Using data from the EPIC Italy cohort, we derived novel DNAm surrogates for BMI, blood pressure, fasting glucose and insulin, cholesterol, triglycerides, and coagulation biomarkers. We validated them in four independent data sets from Europe and the USA. Further, we derived a *DNAmCVDscore* predictive of the time-to-CVD event as a combination of several DNAm surrogates. ROC curve analyses show that *DNAmCVDscore* outperforms previously developed DNAm scores for CVD risk and SCORE2 for short-term CVD risk. Interestingly, the performance of DNAmGrimAge and *DNAmCVDscore* was comparable (slightly lower for DNAmGrimAge, although the differences were not statistically significant).

**Conclusions:**

We described novel DNAm surrogates for CVD risk factors useful for future molecular epidemiology research, and we described a blood DNAm-based composite biomarker, *DNAmCVDscore*, predictive of short-term cardiovascular events. Our results highlight the usefulness of DNAm surrogate biomarkers of risk factors in epigenetic epidemiology to identify high-risk populations. In addition, we provide further evidence on the effectiveness of prediction models based on DNAm surrogates and discuss methodological aspects for further improvements. Finally, our results encourage testing this approach for other NCD diseases by training and developing DNAm surrogates for disease-specific risk factors and exposures.

**Supplementary Information:**

The online version contains supplementary material available at 10.1186/s13148-022-01341-4.

## Background

Emerging epidemiological evidence indicates that composite scores based on blood DNA methylation (DNAm) at different CpG sites are valuable biomarkers to predict complex traits and identify high-risk populations [1–4]. DNAm scores are usually built to model the association of CpG sites with the trait or disease of interest via epigenome-wide association studies (EWAS). However, EWAS suffer from a lack of replication in independent data sets [5], with few exceptions like the well-known DNAm CpGs associated with smoking [6, 7]. Further, it is unclear how the disease risk tracked by DNAm is complementary or redundant with other risk factors for non-communicable diseases (NCDs). In fact, the inclusion of DNAm scores in prediction models often leads to null or marginal prediction improvement compared to traditional models based on classical risk factors like the Framingham Risk Score and SCORE2 for cardiovascular diseases (CVD) [1, 4, 8–10].

In contrast, it has been consistently shown that DNAm scores for estimating individual biological age, named epigenetic clocks [11–15], are associated with several risk factors for NCDs (smoking, alcohol intake, low physical activity, obesity, socio-economic position, and job characteristics) [16–18], and perform very well for predicting ageing-related diseases and all-cause mortality [19, 20]. These results may be explained by how ‘next-generation’ epigenetic clocks like DNAmPhenoAge and DNAmGrimAge have been built [11, 12].

Contrary to classical DNAm scores for NCDs, ‘next-generation’ epigenetic clocks use a two-step approach: (1) development of DNAm surrogates for NCDs risk factors and biomarkers associated with all-cause mortality; (2) development of DNAm epigenetic clocks as a weighted combination of DNAm surrogates. Such a procedure leads DNAm composite scores to be more reliable and reproducible across different cohorts. The best performing epigenetic clock, called DNAGrimAge, incorporates DNAm scores for seven circulating proteins and smoking pack-years, and it has been consistently associated with longevity and numerous age-related diseases, and functional and cognitive outcomes [11, 19, 21]. Other examples of DNAm surrogate of exposures and risk factors include the DNAm biomarkers identified by Colicino and colleagues for cumulative lead exposure [22], the one derived by Marioni and colleagues for several longevity-related and inflammatory proteins [23–26], the classification by Guida and colleagues of current, former (including time since smoking cessation) and never smokers based on blood DNAm biomarkers [7], and the recent characterisation of electronic health records phenotypes by Thompson and colleagues [27].

DNAm surrogates can outperform original exposure measurements in predicting diseases in association studies. For example, Zhang and colleagues show that a combination of smoking-associated DNAm markers predicts lung cancer incidence better than self-reported smoking [28]. In addition, Green and colleagues suggest that a DNAm proxy for C-reactive protein (CRP) predicts structural neuroimaging brain measures better than blood measured CRP [29]. DNAm characteristics can explain these counter-intuitive results: (1) DNAm is a more reliable biomarker than self-reported exposure (i.e. in the case of smoking or other exposures measured through self-reported questionnaires); (2) DNAm variability includes individual genetic and metabolic profiles that can influence individual response to exposure and stressors (i.e. the same amount of exposure can be more or less dangerous based on genetic profile and general state of health); (3) DNAm variations reflect long-term exposures and, in some cases, are more stable in time (e.g. in the case of inflammatory status, the levels of one of the best predictive blood biomarkers, CRP, can fluctuate within a single day).

Because of the way ‘next-generation’ epigenetic clocks have been built (i.e. trained on a set of biomarkers associated with longevity), they are non-specific biomarkers that mirror an individual general state of health rather than the risk for any specific diseases. This study aims to evaluate the possibility of developing disease-specific blood DNAm biomarkers, training a DNAm score on disease-specific exposure and risk factors (rather than on all-cause mortality, as has been done for ‘next-generation’ epigenetic clocks). Specifically, we aim to: (1) develop a DNAm composite biomarker for predicting cardiovascular events trained on CVD-specific risk factors (named *DNAmCVDscore*), and (2) to compare its predictive performance for incident CVD events with (a) the ‘next-generation’ epigenetic clock DNAmGrimAge; (b) a DNAm score for CVD based on a single-step approach developed by Fernández-Sanlés et al., named methylation risk score (MRS) [1]; and (c) a prediction model based on traditional CVD risk factors (chronological age, sex, diabetes status, smoking, systolic blood pressure, total and HDL cholesterol levels), named SCORE2 [10]. Beyond the main study aims, we investigated the association of *DNAmCVDscore* with COVID-19 susceptibility and severity.

## Results

### Study sample and study design description

This study sample includes DNAm data from seven studies previously described [17, 19, 30–35], which is summarised in Table 1.

**Table 1:** Study sample description

Study name	Description	Country	*N*	Age means (min; max)	Female %	Training/Testing set
EPIC Italy	Italian sub-sample of the European Investigation into Cancer and Nutrition study	Italy	1803	53.3 (34.7; 74.9)	62	Training set for DNAm surrogates and *DNAmCVDscore*
EXPOsOMICS CVD	Case–control study on CVD nested in the EPIC Italy cohort	Italy	315	54.9 (35.2; 69.3)	53	Validation set for DNAm surrogates and *DNAmCVDscore*
Understanding Society	The United Kingdom Household Panel Study (UKHLS)	UK	1174	58.0 (28.0; 98.0)	59	Validation set for DNAm surrogates
TILDA	The Irish Longitudinal Study on Ageing	Ireland	490	62.1 (50.0; 80.0)	50	Validation set for DNAm surrogates
GSE174818	Case–control study on COVID-19 susceptibility and progression	USA	127	61.8 (21.0; 90.0)	40	Validation set for DNAm surrogates
NICOLA	The Northern Ireland Cohort for the Longitudinal Study of Ageing	UK	1728	63.99 (40.0; 96.0)	52	Validation set for DNAmCVDscore
HRS	The Health and Retirement Study	USA	2146	68.76 (50.0;100.0)	60	Validation set for DNAmCVDscore

EPIC Italy, the training set, contains 1,803 individuals (62% women), age range from 35 to 75 years, including 295 (16.4%) incident CVD cases [17]. The average (standard deviation sd) time from recruitment to CVD events was 7.6 (3.8) years. The average (sd) follow-up time was 11.3 (5.6) years.

EXPOsOMICS CVD is a case–control study nested in the EPIC Italy cohort, including 160 incident CVD cases and age- and sex-matched controls (not overlapping with EPIC Italy training set), age range from 35 to 70 years (53% women) [30]. The average (sd) time from recruitment to CVD events was 9.6 (3.9) years. The average (sd) follow-up time was 12 (4) years.

TILDA includes data for 490 individuals, originally selected to investigate the association of epigenetic biomarkers of biological ageing with intergenerational socio-economic trajectories, with individuals equally distributed among four socio-economic categories, age range from 50 to 80 years (50% women) [19].

The United Kingdom Household Panel Study (UKHLS), also known as *Understanding Society*, is an ongoing longitudinal, nationally representative study of the UK, designed as a two-stage stratified random sample of the general population. The data used here consist of two pooled cross-sectional waves (waves 2 and 3), age range from 28 to 98 years (59% women) [31].

GSE174818 contains data for 101 COVID-19 cases and 27 age- and sex-matched controls hospitalised with respiratory symptoms, ranging from 21 to 90 years (40% women) [32].

The Northern Ireland Cohort for the Longitudinal Study of Ageing (NICOLA) is a longitudinal cohort representative of the non-institutionalised population of Northern Ireland age 50 years and over [34, 36]. In this study, we used all the individuals with available DNAm data at baseline and follow-up information about cardiovascular events (*N* = 1728; 83 CVD cases). The average (sd) time from recruitment to CVD events was 3.4 (0.4) years. The average (sd) follow-up time was 3.3 (0.3) years.

The Health and Retirement Study (HRS) is a nationally representative longitudinal survey of more than 37,000 individuals over age 50 in 23,000 households in the U.S.A. In this study, we used all the individuals with available DNAm data at baseline and follow-up information about cardiovascular events (*N* = 2146; 209 CVD cases) [37]. The average (sd) time from recruitment to CVD events was 2.9 (1.1) years. The average (sd) follow-up time was 3.6 (0.8) years.

**Fig. 1:**
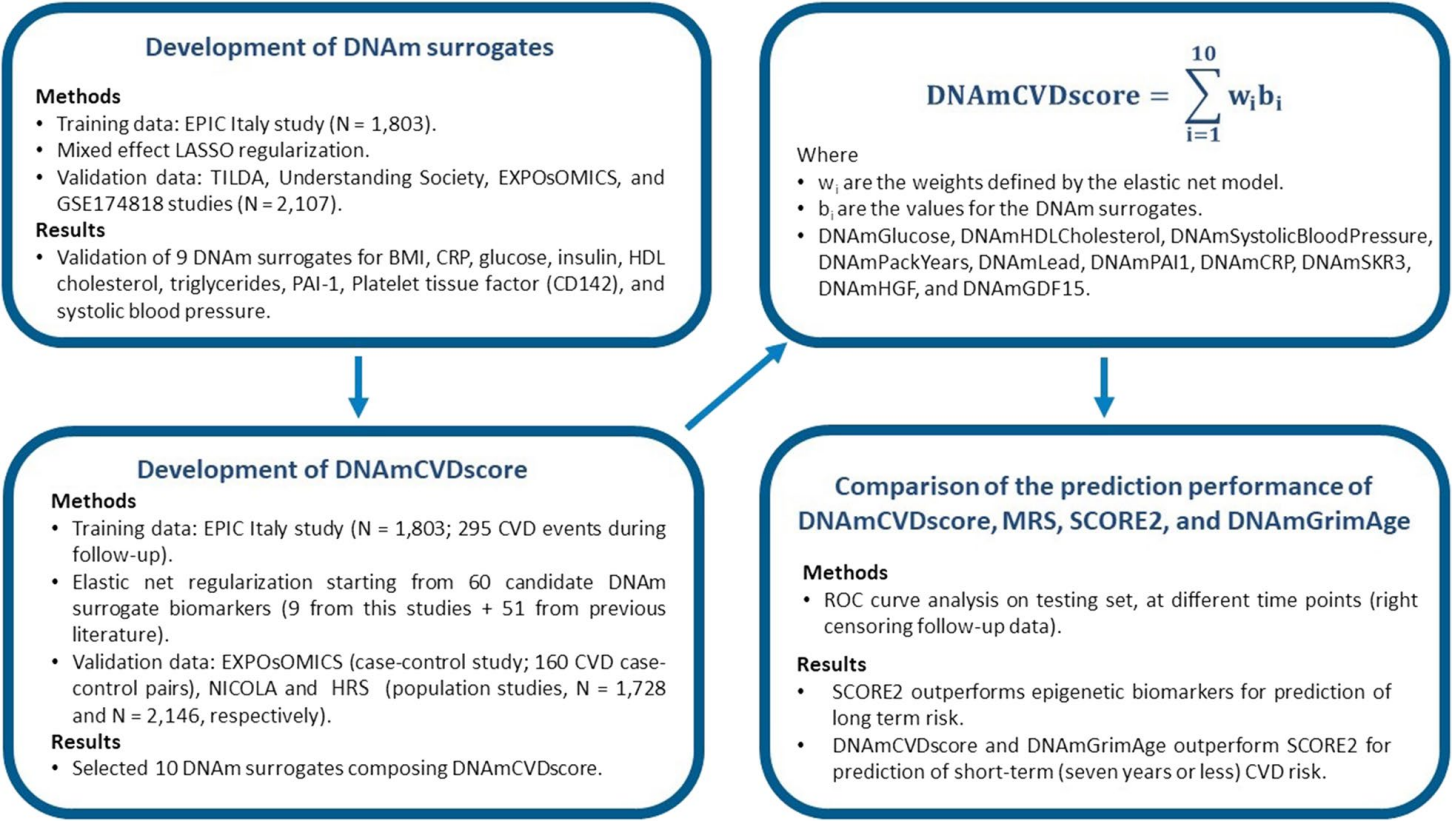
Flow chart for development and validation of *DNAmCVDscore.* Step 1: We train prediction models for developing DNAm surrogates for 13 CVD risk factors/biomarkers using data from the EPIC Italy study (*n* = 1803). We tested the validity of DNAm surrogates in four independent studies (*n* = 2107). Nine out of 13 DNAm biomarkers were validated in the testing set. Step 2: 60 candidate DNAm surrogates (nine newly developed + 51 from the literature) were regressed against the time from study recruitment to cardiovascular event in EPIC Italy (*n* = 1803). The elastic net regression model selected ten DNAm surrogates as components of the *DNAmCVDscore*. Step 3: In EXPOsOMICS CVD data set (*N* = 315), NICOLA (N = 1728), and HRS (*N* = 2146) we evaluated the prediction performance of *DNAmCVDscore* at different time points (right-censoring follow-up time) using logistic regression models adjusted for chronological age, sex, and recruitment centre (matching variables in EXPOsOMICS CVD) or Cox regression models (in NICOLA and HRS). *DNAmCVDscore* has a higher AUC for short-term cardiovascular events than for long-term CVD. Step 4: We compared the prediction performance of *DNAmCVDscore* with previously developed composite biomarkers: MRS, DNAmGrimAge, SCORE2 and SCORE2 + *DNAmCVDscore*. SCORE2 outperforms epigenetic predictors for long-term CVD risk (occurred more than 8 years after recruitment), whereas *DNAmCVDscore* predicts short-term events (occurred within 7 years after recruitment) better than other biomarkers. The enriched SCORE2 + *DNAmCVDscore* model outperformed all the competitors for the entire time horizon considered in the study

In Fig. 1, we present the analytical flow chart summarising the main steps for developing the *DNAmCVDscore*:

1. Develop and validate novel DNAm surrogate biomarkers (training set: EPIC Italy; testing sets: EXPOsOMICS CVD, Understanding Society, TILDA, and GSE174818) through LASSO regularisation for linear regression model.
2. Develop the *DNAmCVDscore* (training set: EPIC Italy; 60 candidate DNAm surrogate biomarkers) through elastic net for Cox proportional hazards model.
3. Validation of the *DNAmCVDscore* investigating its prediction performance through ROC curve analysis, right-censoring follow-up data at different time points in EXPOsOMICS CVD data set, and through C-index in NICOLA and HRS data sets.
4. Comparison of *DNAmCVDscore*, MRS, SCORE2, and DNAmGrimAge predictive value.

### Estimation and validation of DNAm surrogates

By means of penalised linear regression models (see “Methods” section), we developed DNAm surrogates for body mass index (BMI), systolic and diastolic blood pressure, and ten blood measured biomarkers: total cholesterol, HDL cholesterol, LDL cholesterol, triglycerides, plasminogen activator inhibitor-1 (PAI-1), C-reactive protein (CRP), D-dimer, platelet tissue factor (a.k.a. CD142 protein), fasting glucose, and insulin.

A 75% proportion of the EPIC Italy data set (*N* = 1352) was employed as training set, the remaining 25% as a primary testing set, while the other four studies (EXPOsOMICS CVD, Understanding Society, TILDA, and GSE174818) were considered for validation. In Table 2, we report the number of CpGs whose linear combination best predicted the corresponding marker and the Pearson correlation coefficients of observed (measured) *vs* predicted (DNAm surrogate) in the EPIC Italy testing set (25% of the total sample). The correlation of DNAm surrogates with the corresponding measured marker was always higher than 0.4 (all *P* values lower than 0.0001), ranging from 0.43 (DNAmPAI-1 vs PAI-1) to 0.73 (DNAmTriglycerides vs triglycerides). Further, in Table 2, we report the Pearson correlation coefficients of observed *vs* predicted values computed in the four validation data sets. The correlation of DNAm surrogates with the corresponding measured marker was always positive, ranging from 0.08 (DNAmHDL *vs* HDL cholesterol) to 0.44 (DNAmInsulin vs insulin). The *P* value was lower than 0.05 for all but D-dimer, diastolic blood pressure, LDL cholesterol, and total cholesterol. Based on the above, we validated nine (out of 13) DNAm surrogates for BMI, CRP, fasting glucose and insulin, HDL cholesterol, triglycerides, PAI-1, platelet tissue factor (CD142), and systolic blood pressure. In Additional file 1: Figure S1, we reported the scatterplots of the standardised observed *vs* predicted values for the nine DNAm surrogates validated in this study.

**Table 2:** List of newly developed DNAm surrogate biomarkers

Model training, EPIC Italy training set, *n* = 1352			Results on EPIC ITALY test set *n* = 451		Results on the validation set			
Risk factor/biomarker	Model type	Number of CpGs	Pearson R	*P*	Validation data sets (N)	Pearson R	*P*	Validated DNAm surrogate
BMI	Mixed-effect LASSO	405	0.59	< 0.0001	US, TILDA, EXPOsOMICS, GSE174848 (2,045)	0.27	< 0.0001	Yes
CRP	LASSO	265	0.57	< 0.0001	US, TILDA, EXPOsOMICS, GSE174849 (1,893)	0.23	< 0.0001	Yes
D-dimer	LASSO	483	0.72	< 0.0001	EXPOsOMICS, GSE174848 (248)	0.17	0.56	No
Diastolic blood pressure	Mixed-effect LASSO	401	0.57	< 0.0001	EXPOsOMICS, TILDA (772)	0.10	0.36	No
Glucose	Mixed-effect LASSO	354	0.67	< 0.0001	EXPOsOMICS, TILDA, US (1,810)	0.28	0.007	Yes
HDL cholesterol	Mixed-effect LASSO	151	0.58	< 0.0001	EXPOsOMICS, TILDA, US (1,829)	0.08	0.001	Yes
Insulin	Mixed-effect LASSO	574	0.66	< 0.0001	EXPOsOMICS (170)	0.44	< 0.0001	Yes
LDL cholesterol	Mixed-effect LASSO	368	0.62	< 0.0001	EXPOsOMICS, TILDA (661)	0.15	0.36	No
PAI-1	LASSO	90	0.43	< 0.0001	EXPOsOMICS (171)	0.28	0.0001	Yes
Systolic blood pressure	Mixed-effect LASSO	275	0.64	< 0.0001	EXPOsOMICS, TILDA (772)	0.28	0.001	Yes
Tissue factor (CD142)	Mixed-effect LASSO	197	0.62	< 0.0001	EXPOsOMICS (171)	0.16	0.03	Yes
Total cholesterol	Mixed-effect LASSO	257	0.53	< 0.0001	EXPOsOMICS, TILDA, US (1,830)	0.13	0.14	No
Triglycerides	LASSO	471	0.73	< 0.0001	EXPOsOMICS, TILDA (661)	0.22	0.0003	Yes

For each candidate marker, we reported: the model used to extract significant CpGs (LASSO or mixed-effect LASSO depending on the association with the centre of recruitment), the number of CpGs whose linear combination constitute the best marker prediction, the Pearson correlation coefficient and p value in the primary test set (random 25% of EPIC Italy samples), the Pearson correlation coefficient and p value in independent test sets (random effect meta-analysis across studies). Nine out of 13 DNAm surrogates for CVD risk factors/markers were validated in independent testing set (*P* value for the Pearson correlation test lower than 0.05). The lists of CpGs and their weights to compute DNAm surrogates in independent data sets are provided in Additional file 1

#### Comparison with previously developed DNAm surrogates

We compared our newly developed DNAm surrogates with previously developed DNAm surrogates for HDL cholesterol, BMI [23], and PAI-1 [11]. The Pearson correlation coefficients of our DNAm surrogates with those previously developed were 0.31 (*P* < 0.0001), 0.45 (*P* < 0.0001), and 0.36 (*P* < 0.0001) for HDL cholesterol, BMI, and PAI-1, respectively.

#### Development and validation of the DNAmCVDscore

We developed a combined score, *DNAmCVDscore*, predictive of future CVD events by regressing the time-to-CVD event on 60 candidate DNAm surrogates: the nine newly developed within this study, 32 DNAm surrogates for blood measured (mainly inflammatory) proteins produced by Gadd and colleagues [23, 26]; three epigenetic clocks (HorvathDNAmAge, HannumDNAmAgem, and DNAmPhenoAge) [20]; two DNAm surrogates for lead exposure [22]; six ‘Houseman’ DNAm surrogates for white blood cell (WBC) proportion [38]; and the nine components of the DNAmGrimAge clock (DNAm surrogates for smoking pack-years, telomere length, and seven blood measured proteins) [11].

The elastic net Cox regression model employed for the purpose selected chronological age, sex, and DNAm surrogates for blood measured glucose, HDL cholesterol, systolic blood pressure, PAI-1, CRP (developed within this study), Serine/threonine-protein kinase receptor 3 (SKR3) and hepatocyte growth factor (HGF) (developed in Gadd et al. [25]), growth differentiation factor 15 (GDF15) protein, smoking pack-years (developed in Lu et al. [11]), and lead level measured in patella bone (developed in Colicino et al. [22]). Since age and sex effects are considered in several DNAm surrogate biomarkers that were used to derive the *DNAmCVDscore* (Glucose, PAI-1, SBP, CRP, HDL, GDF15, and PACKYRS) we re-trained the elastic net model without age and sex to derive better calibrated coefficients (not influenced by the redundant presence of age and sex). All the biomarkers except DNAmHDL have positive regression coefficients (higher risk associated with higher values). The linear combination of standardised values for the ten DNAm surrogates listed in Table 3 can be interpreted as a standardised (within the population in which it is computed) CVD risk score (named *DNAmCVDscore*). In Additional file 1: Figure S2, we report the pairwise Pearson’s correlation coefficients for the 10 DNAm surrogates used to derive the *DNAmCVDscore*.

**Table 3:** DNAm surrogates composing the DNAmCVD score

Study	DNAm surrogate biomarker	*DNAmCVDscore* coefficient	Original biomarker/risk factor
This study	DNAmGlucose	0.0329	Blood glucose
This study	DNAmHDL	− 0.4473	Blood HDL cholesterol
This study	DNAmSBP	0.1420	Systolic blood pressure
This study	DNAmCRP	0.0276	Blood C-reactive protein
This study	DNAmPAI1	0.1679	Blood Plasminogen activator inhibitor 1
Gadd et al. 2022	DNAmSKR3	0.0362	Blood Serine/threonine-protein kinase receptor R3
Gadd et al. 2022	DNAmHGF	0.0371	Blood Hepatocyte growth factor
Colicino et al. 2021	DNAmLeadPatella	0.0402	Lead levels in Patella’s bone
Lu et al. 2019	DNAmGDF15	0.0947	Blood Growth Differentiation Factor 15
Lu et al. 2019	DNAmPACKYRS	0.1192	Smoking pack-years

*DNAmCVDscore* is computed as a linear combination of standardised (mean = 0, variance = 1) DNAm surrogates with weights listed in the coefficient column. All biomarkers but DNAmHDL have positive coefficients (higher CVD risk associated with a higher value for the biomarker)

For validating the *DNAmCVDscore,* we used two different analytical approaches, depending on the study design of the validation data sets. The EXPOsOMICS CVD study had a nested case–control study design, with cases matched to healthy controls for chronological age, sex, recruitment centre, and technical covariates (controls selected using the incidence sampling method). Therefore, we test the *DNAmCVDscore* prediction performance using logistic regression models adjusted for matching variables. By contrast, NICOLA and HRS data sets include data from the general population over the age of 50 from Northern Ireland and the U.S.A., respectively (cohort study design), so we used Cox proportional hazard regression models.

Also, in the independent test sets we compared *DNAmCVDscore* predictive performance with those of MRS, SCORE2, and DNAmGrimAge. In EXPOsOMICS CVD, we performed ROC curve analyses of logistic regression models adjusted for matching variables, right-censoring the follow-up at constant intervals of one year from 18 to 2 years to evaluate the prediction performance as a function of the follow-up time. In NICOLA and HRS, because of the different study design (cohort studies) and the lower follow-up time (up to 5 years, 3 years on average), we computed the Harrell’s concordance index (C-index) from Cox proportional hazard regression models. Further, we have evaluated the prediction performance of a model including the *DNAmCVDscore* in addition to the traditional risk factors in SCORE2 (named from here on SCORE2 + *DNAmCVDscore* model).

#### Validation of DNAmCVDscore in EXPOSOMICS CVD

**Fig. 2:**
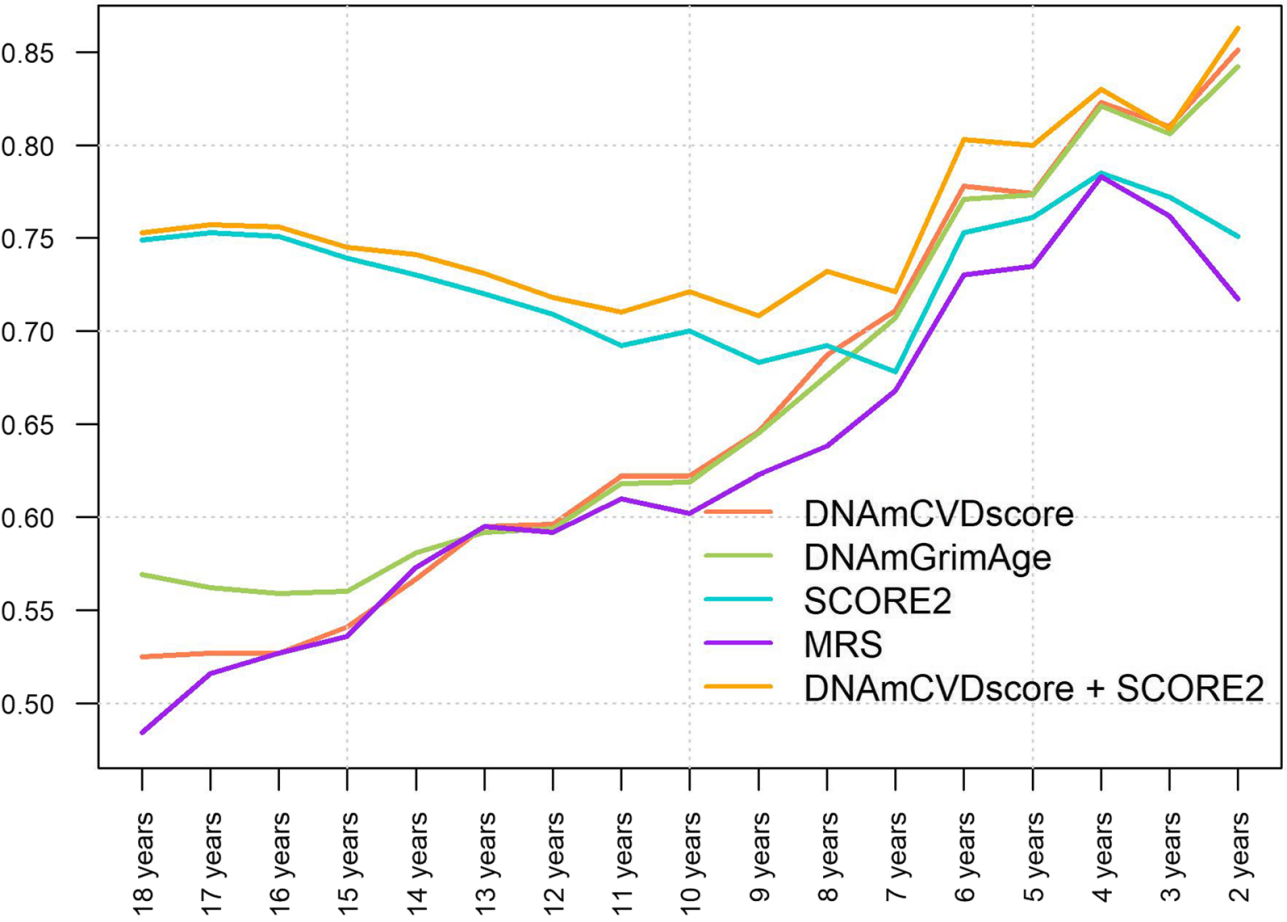
Prediction performance of *DNAmCVDscore*, MRS, DNAmGrimAge, SCORE2 and SCORE2 + *DNAmCVDscore*. Area under the ROC curve (AUC), on the y-axis, as a function of the follow-up length (*x*-axis) for the five composite biomarkers investigated in this study. MRS has the worst prediction performance at each time point. SCORE2 outperforms epigenetic predictors for long-term CVD risk (occurred more than 8 years after recruitment), whereas *DNAmCVDscore* and DNAmGrimAge predict short-term risk (CVD events within 7 years after recruitment or less) better than the other biomarkers. The enriched SCORE2 + *DNAmCVDscore* model outperformed all the competitors for the entire time horizon considered in the study

In Table 4 and Fig. 2, we present the area under the ROC curve (AUC), sensitivity, and specificity (best threshold selected according to the minimum distance from the top left corner of the ROC curve) of the five composite biomarkers as a function of the length of follow-up (right censored at regular intervals of one year) in EXPOsOMICS CVD data set. For all models regressed only on epigenetic-based biomarkers, namely *DNAmCVDscore*, MRS, and DNAmGrimAge, the AUC increases as the follow-up time decreases, suggesting that epigenetic biomarkers predict short-term events rather than long-term CVD risk (Table 4 and Fig. 2). By contrast, the AUC for SCORE2 was not time-dependent, ranging from 0.678 (7 years follow-up) to 0.785 (4 years follow-up). The MRS had the worst performance independently of the follow-up length (Table 4 and Fig. 2). SCORE2 outperformed epigenetic biomarkers in predicting CVD events considering follow-up time from 18 to 8 years. However, right-censoring the follow-up time at 7 years or less, *DNAmCVDscore* and DNAmGrimAge perform better than SCORE2, with *DNAmCVDscore* having a slightly higher AUC than DNAmGrimAge (Table 4 and Fig. 2). Overall, the best performance throughout the considered time horizon is showcased by the SCORE2 + *DNAmCVDscore* model, in which the original SCORE2 model is enriched with the *DNAmCVDscore* developed in this study. Epigenetic-based biomarkers improve the predictive accuracy of SCORE2, complementing the information provided by traditional CVD risk factors. This is particularly true when a short follow-up time is considered, where the differences between SCORE2 and SCORE2 + *DNAmCVDscore* are evident.

**Table 4:** Results from the ROC curve analyses in EXPOsOMICS CVD

Follow-up time	# Events	*DNAmCVDscore*		DNAmGrimAge		SCORE2		MRS		SCORE2 + DNAmCVDscore	
		AUC (95% CI)	Sensitivity; specificity	AUC (95% CI)	Sensitivity; specificity	AUC (95% CI)	Sensitivity; specificity	AUC (95% CI)	Sensitivity; specificity	AUC (95% CI)	Sensitivity; specificity
18 years	160	0.525 (0.461; 0.589)	0.477; 0.580	0.569 (0.505; 0.632)	0.647; 0.482	0.749 (0.696; 0.803)	0.719; 0.698	0.484 (0.420; 0.548)	0.523; 0.500	0.753 (0.700; 0.807)	0.732; 0.685
17 years	159	0.527 (0.463; 0.591)	0.520; 0.553	0.562 (0.498; 0.626)	0.669; 0.472	0.753 (0.700; 0.806)	0.753; 0.671	0.516 (0.452; 0.580)	0.545; 0.484	0.757 (0.704; 0.810)	0.740; 0.689
16 years	158	0.527 (0.463; 0.591)	0.513; 0.554	0.559 (0.496; 0.623)	0.583; 0.572	0.751 (0.698; 0.805)	0.718; 0.692	0.527 (0.463; 0.591)	0.449; 0.610	0.756 (0.704; 0.809)	0.705; 0.717
15 years	149	0.541 (0.477; 0.604)	0.518; 0.577	0.560 (0.496; 0.623)	0.681; 0.450	0.739 (0.684; 0.794)	0.741; 0.671	0.536 (0.472; 0.600)	0.509; 0.477	0.745 (0.691; 0.799)	0.687; 0.732
14 years	139	0.567 (0.504; 0.630)	0.483; 0.662	0.581 (0.518; 0.644)	0.494; 0.662	0.730 (0.674; 0.786)	0.705; 0.691	0.573 (0.509; 0.636)	0.682; 0.475	0.741 (0.686; 0.796)	0.688; 0.683
13 years	123	0.595 (0.531; 0.659)	0.625; 0.537	0.592 (0.528; 0.656)	0.526; 0.667	0.720 (0.663; 0.777)	0.662; 0.699	0.595 (0.531; 0.659)	0.531; 0.650	0.731 (0.676; 0.787)	0.651; 0.740
12 years	111	0.596 (0.530; 0.662)	0.529; 0.649	0.594 (0.529; 0.659)	0.510; 0.694	0.709 (0.650; 0.768)	0.588; 0.775	0.592 (0.525; 0.658)	0.588; 0.613	0.718 (0.661; 0.775)	0.598; 0.757
11 years	95	0.622 (0.554; 0.690)	0.536; 0.705	0.618 (0.549; 0.686)	0.568; 0.663	0.693 (0.630; 0.755)	0.582; 0.758	0.610 (0.540; 0.680)	0.536; 0.695	0.710 (0.651; 0.770)	0.600; 0.737
10 years	84	0.622 (0.551; 0.693)	0.580; 0.607	0.619 (0.548; 0.691)	0.576; 0.631	0.700 (0.636; 0.765)	0.550; 0.845	0.602 (0.530; 0.673)	0.541; 0.667	0.721 (0.661; 0.781)	0.554; 0.821
9 years	67	0.647 (0.571; 0.722)	0.601; 0.612	0.645 (0.569; 0.721)	0.649; 0.582	0.683 (0.611; 0.754)	0.609; 0.746	0.623 (0.546; 0.700)	0.581; 0.627	0.708 (0.642; 0.775)	0.577; 0.731
8 years	55	0.687 (0.611; 0.762)	0.569; 0.709	0.676 (0.601; 0.752)	0.627; 0.673	0.692 (0.618; 0.766)	0.623; 0.746	0.638 (0.554; 0.722)	0.635; 0.618	0.732 (0.664; 0.800)	0.612; 0.782
7 years	37	0.711 (0.626; 0.796)	0.615; 0.703	0.707 (0.625; 0.789)	0.554; 0.811	0.678 (0.587; 0.770)	0.622; 0.703	0.668 (0.566; 0.770)	0.669; 0.622	0.721 (0.641; 0.801)	0.741; 0.649
6 years	28	0.778 (0.702; 0.854)	0.673; 0.786	0.771 (0.688; 0.853)	0.634; 0.821	0.753 (0.668; 0.839)	0.686; 0.786	0.730 (0.616; 0.844)	0.669; 0.750	0.803 (0.740; 0.867)	0.676; 0.893
5 years	23	0.774 (0.695; 0.853)	0.623; 0.826	0.773 (0.694; 0.851)	0.634; 0.826	0.761 (0.665; 0.856)	0.723; 0.783	0.735 (0.618; 0.852)	0.688; 0.783	0.800 (0.741; 0.860)	0.757; 0.783
4 years	16	0.823 (0.746; 0.899)	0.799; 0.688	0.821 (0.736; 0.906)	0.632; 0.938	0.785 (0.653; 0.917)	0.722; 0.875	0.783 (0.649; 0.917)	0.689; 0.875	0.830 (0.755; 0.906)	0.773; 0.750
3 years	13	0.811 (0.720; 0.901)	0.642; 0.846	0.806 (0.724; 0.888)	0.652; 0.846	0.772 (0.620; 0.923)	0.772; 0.846	0.762 (0.628; 0.896)	0.768; 0.769	0.809 (0.726; 0.892)	0.705; 0.846
2 years	7	0.851 (0.767; 0.935)	0.799; 0.857	0.842 (0.745; 0.939)	0.737; 0.857	0.751 (0.575; 0.926)	0.656; 0.857	0.717 (0.562; 0.872)	0.779; 0.571	0.863 (0.766; 0.960)	0.766; 0.857

For each composite biomarker, we report the AUC (95% CI), sensitivity, and specificity according to the best threshold (minimising the distance from the top left corner of the ROC curve) derived from logistic regression model adjusted for matching parameters (age, sex, and centre of recruitment). Predictive performance was evaluated at different time points, right-censoring the follow-up time in the range of 18 to two years, with one year interval

#### Validation of DNAmCVDscore in NICOLA and HRS

In Cox regression models adjusted for age and sex, the hazard ratio (HR) for one standard deviation increase in the DNAmCVDscore was 2.05 (95% CI 1.13; 3.73, *P* = 0.02) and 1.70 (95% CI 1.12; 2.58, *P* = 0.01) in NICOLA and HRS data sets, respectively. In Table 5, we reported the C-index and their 95% confidence intervals for the five composite biomarkers (*DNAmCVDscore*, DNAmGrimAge, MRS, SCORE2, and SCORE2 + *DNAmCVDscore*). In line with EXPOsOMICS CVD, *DNAmCVDscore* performed slightly better than DNAmGrimAge and SCORE2, whereas MRS had the poorest predictive performance. Finally, the combined model including SCORE2 variables + *DNAmCVDscore* had a slightly higher C-index than the model with *DNAmCVDscore* alone.

**Table 5:** Results from the Cox (proportional hazard) regression models in NICOLA and HRS validation data sets

	NICOLA (*N* = 1728; 83 CVD cases)	HRS (*N* = 2146; 209 CVD cases)
	C-index (95% CI)	C-index (95% CI)
*DNAmCVDscore*	0.73 (0.68; 0.78)	0.70 (0.65; 0.73)
DNAmGrimAge	0.72 (0.67; 0.77)	0.70 (0.65; 0.74)
SCORE2	0.72 (0.67; 0.77)	0.69 (0.65; 0.74)
MRS	0.69 (0.64; 0.75)	0.68 (0.63; 0.72)
*DNAmCVDscore* + SCORE2	0.75 (0.69; 0.80)	0.71 (0.67; 0.75)

For each composite biomarker, we report the C-index (95% CI) derived from Cox regression models adjusted for age and sex

We performed additional statistical comparisons, including sensitivity analyses, correlation of *DNAmCVDscore* with epigenetic clocks, and the association of *DNAmCVDscore* with COVID-19 case–control status and severity. The results of these additional analyses are reported in Additional file 1.

## Discussion

Emerging evidence highlights the epidemiological value of composite scores based on blood DNAm surrogates of exposures and risk factors, e.g. epigenetic clocks, associated with non-communicable diseases (NCDs) and predictive of mortality [20]. However, since ‘next-generation’ epigenetic clocks have been trained on time to death, they constitute non-specific biomarkers, representative of the general individual state of health, rather than disease-specific biomarkers.

In this work, we present a combined blood DNAm-based biomarker for predicting future cardiovascular events, named *DNAmCVDscore*. To the best of our knowledge, this is the first example of a disease-specific biomarker using molecular data only, without the need for additional information (other than age and sex) about the personal history of exposure, general state of health, lifestyle habits, and other commonly used biomarkers. This may be important for future risk prediction reducing the scope of measurement error and bias attendant on self-reports of exposure to risk factors. For this aim, DNAm-based biomarkers are optimal candidates because DNAm is strongly influenced by long-term exposures, genetic susceptibility, and lifestyle habits [39]. In other words, it is possible to extract information about the history of exposures and susceptibility to complex diseases from whole-genome DNAm data with high accuracy.

We applied a two-step approach, following the successful example of the epigenetic clocks. First, we developed and validated nine novel DNAm surrogates for CVD risk factors: systolic blood pressure, BMI, CRP, fasting glucose and insulin, HDL cholesterol, triglycerides, PAI-1, and platelet tissue factor (a.k.a. CD142 protein). In Additional file 1, we provided the lists of CpG sites and their weights for generating the new DNAm surrogates in independent data sets for future epidemiological research.

Then, we developed a *DNAmCVDscore* starting from 60 candidate DNAm surrogates (nine newly developed within this study plus 51 from the previous literature), including surrogate measures for the main risk factors for CVDs (obesity, smoking habits, alcohol consumption, inflammatory proteins, lipid levels, blood pressure, coagulation biomarkers). Our elastic net model extracted ten DNAm surrogate biomarkers whose linear combination constitutes the so-called *DNAmCVDscore*: fasting glucose, HDL cholesterol, systolic blood pressure, smoking pack-years, lead exposure and blood levels of PAI-1, CRP, SKR3, HGF, and GDF15 proteins.

We validated the ability of the *DNAmCVDscore* to predict future cardiovascular events in an independent prospective case–control study nested in the EPIC Italy cohort (EXPOsOMICS CVD) and two cohort studies from Northern Ireland and the U.S.A. (NICOLA and HRS). EXPOsOMICS CVD matched incident CVD cases with healthy controls by age, sex, recruitment centre, and length of follow-up (up to 18 years; around 12 years on average) using the incident density sampling method, while NICOLA and HRS are cohort studies from the general population over the age of 50 with a follow-up time up to 5 years (3 years on average). We showed that existing prediction models based on traditional CVD risk factors (SCORE2 [10], based on chronological age, sex, diabetes, smoking, systolic blood pressure, total and HDL cholesterol) outperform epigenetic biomarkers for predicting long-term CVD risk according to the AUC measure. However, *DNAmCVDscore* predicts short-term (7 years follow-up or less) CVD risk better than SCORE2. Other known CVD scores based on traditional risk factors like the Framingham Risk Score (FRS) [40], share the majority of predictors with SCORE2, consequently, they have prediction performance comparable to that of SCORE2 (data not shown to avoid redundancy).

When traditional risk factors are combined with epigenetic biomarkers, as done in the SCORE2 + *DNAmCVDscore* model, the best performance for both long- and short-term CVD risk is achieved. Interestingly, the prediction performance of *DNAmCVDscore* and DNAmGrimAge was comparable (slightly higher for *DNAmCVDscore* for short-term events). Accordingly, in Additional file 2: Table S2 we showed that the combination SCORE2 + DNAmGrimAge was comparable (slightly lower) to the combination *SCORE2* + *DNAmCVDscore,* and that the combination SCORE2 + *DNAmCVDscore* + DNAmGrimAge does not outperform SCORE2 + *DNAmCVDscore,* further supporting that *DNAmCVDscore* and DNAmGrimAge shared a significant proportion of variability. This is not unexpected considering the *DNAmCVDscore* and DNAmGrimAge share four of the ten components (DNAmCRP, DNAmPAI1, DNAmPackYears, and DNAmGDF15), and the Pearson correlation coefficient for both epigenetic biomarkers is *R* = 0.56 (*P* < 0.0001, Additional file 1: Figure S4). Such similarities may be explained by the fact that CVD is the leading cause of mortality worldwide and that the DNAmGrimAge was trained to predict time to death in the Framingham Heart Study, in which there is detailed characterisation and documentation of heart disease [11]. From a biological perspective, these results confirm previous research indicating that heightened inflammation (associated with all four components common in both scores) plays a major role in biological ageing and the risk of age-related diseases, including CVDs [41].

Finally, we showed that the MRS, built directly from modelling the association of CpGs on CVD risk using a single-step approach, had the worst prediction performance independently of the length of follow-up and study design.

Among the 10 DNAm surrogates used to derive the *DNAmCVDscore,* DNAmHDL had the largest absolute coefficient, consistent with the strong association of serum HDL with CVD risk in the training set, which explains more than 65% of the variability in *DNAmCVDscore* (Pearson correlation coefficient between DNAmHDL and *DNAmCVDscore* = − 0.82, Additional file 2: Table S2). As such, we performed additional sensitivity analyses to evaluate the prediction performance of DNAmHDL alone. In the testing sets, the prediction of *DNAmCVDscore* was higher than DNAmHDL, suggesting that each of the 10 surrogate biomarkers captures a slice of the variability in the risk of future CVD events. Also, the pairwise correlation coefficients among the 10 surrogates were generally lower than 0.3 with few exceptions (high mutual correlation between DNAmPACKYRS, DNAmHGF, and DNAmSKR3, Additional file 2: Table S2), further supporting previous interpretation.

The results described above suggest that blood DNAm predictor of diseases may be improved. For example, the *DNAmCVDscore* can be ameliorated in different ways:

1. More DNAm surrogates, such as surrogate measures for air pollution exposure, physical activity, dietary quality (e.g. adherence to the Mediterranean diet or consumption of ultra-processed food) [30, 42–44] should be developed and included among the list of candidates in the training model.
2. Refined statistical methods can be used to improve DNAm biomarkers reproducibility and reducing noise due to unmeasured batch effect [45, 46] and to evaluate their predictive performance for multiple outcomes [47]. Given the likely life course effects on methylation trajectories, alternative functional forms other than linear combinations should also be explored possibly using longitudinal data.
3. Acknowledging the drawbacks of shrinkage methods like the elastic net [48], we should look towards increasing the sample size of the training set by combining data from multiple cohorts and different countries, possibly modelling country-specific risk factors to improve results generalisability.
4. A more comprehensive evaluation of their real-world value, incorporating calibration, clinical utility, and net benefit [49].

Also, we showed that, although *DNAmCVDscore* is not directly trained on age, it is correlated with chronological age (*R* = 0.41, *P* < 0.0001) and epigenetic clocks (Additional file 1: Figure S2). These results further support the idea that susceptibility due to increasing ageing is included in the *DNAmCVDscore*, even if chronological age (or epigenetic clocks) does not directly contribute to it.

Further, we demonstrated the usefulness of DNAm surrogate biomarkers in investigating COVID-19 susceptibility and severity, showing that *DNAmBMI* was associated with case–control status, while measured BMI was not (Additional file 1; Additional file 2: Table S2), and that *DNAmCRP* outperformed blood measured CRP in predicting disease severity (Additional file 1; Additional file 2: Table S2). Finally, we showed that *DNAmCVDscore* is higher in COVID-19 patients than in controls (hospitalised with respiratory problems) and that a higher *DNAmCVDscore* is associated with a worse prognosis (according to the GRAM score) after COVID-19 infection (Additional file 1: Figure S4). These results support recent literature suggesting COVID-19 shares direct and indirect determinants (i.e. ethnicity, socio-economic status) with other NCDs, supporting the concept of COVID-19 as a syndemic [50, 51] with implications for restrictions and prevention strategies.

This work has limitations. The training set for the time from recruitment to the cardiovascular events comes from the Italian population, and the predictive performance for long-term CVD was poor. This result is partially explained by our selection procedure in the training set, based on the time-to-CVD event. Also, it may support a previous report about DNAm as biomarkers of life course accumulation of exposure and stressors [52], leading to a better prediction of short-term outcomes rather than long-term risk. Thus, methylation levels are likely to have undergone severe changes as the follow-up time increased. Moreover, in the current study, incident CVD events many years after recruitment were mostly limited to individuals with baseline diabetes, an aspect taken into account by traditional scores based on risk factors but not included in our DNAm-based score.

We discussed previously how *DNAmCVDscore* could be refined by re-training the model after increasing the sample size and using updated analytical methods.

## Conclusions

We developed a combined biomarker as a linear combination of DNAm surrogates, named *DNAmCVDscore*, with high performance in predicting short-term cardiovascular events outperforming current state-of-the-art CVD prediction models based on traditional risk factors, and DNAm scores based on a single-step approach. Further, we provided new DNAm surrogates for CVD risk factors useful for further research in molecular epidemiology.

This work provides a proof of concept about the effectiveness of the described methodology based on a two-step approach which involve DNAm surrogates. Developing blood-based biomarker for risk prediction without the need for additional information or invasive measurements would provide significant opportunities to reduce disease burden from a public health perspective.

Our results encourage further studies investigating the association of the newly developed *DNAmCVDscore* with secondary outcomes that result from CVD (such as lung function, reduced cognitive and mobility outcomes), and to test this two-step approach for other NCD diseases (such as cancer, mental diseases, neurodegenerative diseases, respiratory problems, and hearing and taste loss) by training and developing DNAm surrogates for disease-specific risk factors and exposures.

## Methods

### Subject recruitment, demographic/lifestyle variables acquisition, and DNA methylation measurements

#### EPIC Italy

Study participants were drawn from the Italian component of the European Prospective Investigation into Cancer and Nutrition (EPIC) cohort [53], a large general population cohort consisting of ~ 520,000 individuals, with standardised lifestyle and personal history questionnaires, measured anthropometric data and blood samples collected for DNA extraction. Smoking habits data were collected at study enrolment using a questionnaire, and participants were categorised as ‘never’, ‘former’ and ‘current’ smokers. Height and weight were measured at enrolment with a standardised protocol, and body mass index (BMI) was calculated as the ratio between weight in kg and squared height in metres, treated as a continuous variable. Measurements methods for blood pressure, cholesterol levels, triglycerides, and PAI1, D-dimer, and CRP are reported elsewhere [54].

This study sample includes individuals from five nested case–control studies on breast, colon, and lung cancer, lymphomas, and myocardial infarction [55, 56]. Participants were sampled from the 47,749 participants of the EPIC Italy cohort and included 354 incident breast cancer cases, 169 incident colon cancer cases, 192 incident lung cancer cases, 72 incident lymphoma cases, 295 incident myocardial infarction cases and their 1079 matched controls. Controls were individually matched on age (± 5 years), sex, the season of blood collection, centre, and length of follow-up. Since the disease diagnoses were made years after the blood draw, all the subjects were treated as healthy at recruitment. In the time-to-CVD event analyses, the follow-up time was (right) censored at the time of diagnosis for incident cancer cases. Overall, after DNA methylation data quality controls and sample filtering 1,803 EPIC Italian subjects were used in this analysis.

#### EXPOsOMICS CVD

Study participants pertain to the EPIC Italy cohort. 160 incident CVD cases and one-to-one matched controls (not overlapping with the EPIC Italy data set described hereafter) were extracted using the incident density sampling method [30]. After DNAm data quality control and sample filtering, 315 individuals were included in this study.

For the microarray (in EPIC Italy and EXPOsOMICS CVD), DNA samples were extracted from buffy coats using the QIAsymphony DNA Midi Kit (Qiagen, Crawley, UK). Bisulphite conversion of 500 ng of each sample was performed using the EZ-96 DNA Methylation-Gold™ Kit according to the manufacturer’s protocol (Zymo Research, Orange, CA). Then, bisulphite-converted DNA was used for hybridisation on the Infinium HumanMethylation450 BeadChip, following the Illumina Infinium HD Methylation protocol. Briefly, a whole-genome amplification step was followed by enzymatic end-point fragmentation and hybridisation to HumanMethylation 450 BeadChips at 48 °C for 17 h, followed by single nucleotide extension. The incorporated nucleotides were labelled with biotin (ddCTP and ddGTP) and 2,4-dinitrophenol (DNP) (ddATP and ddTTP). After the extension step and staining, the BeadChip was washed and scanned using the Illumina HiScan SQ scanner. The intensities of the images were extracted using the GenomeStudio (v.2011.1) Methylation module (1.9.0) software, which normalises within-sample data using different internal controls that are present on the HumanMethylation 450 BeadChip and internal background probes. The methylation score for each CpG was represented as a β value according to the fluorescent intensity ratio representing any value between 0 (unmethylated) and 1 (completely methylated).

#### The Irish longitudinal study on ageing (TILDA)

Is a large prospective cohort study examining the social, economic and health circumstances of 8,175 community-dwelling older adults aged 50 years and over resident in the Republic of Ireland. The sample was generated using a 3-stage selection process and the Irish Geodirectory as the sampling frame. The Irish Geodirectory is a comprehensive listing of all addresses in the Republic of Ireland, which is compiled by the national post service and ordnance survey Ireland. Subdivisions of district electoral divisions pre-stratified by socio-economic status, age, and geographical location, served as the primary sampling units. The second stage involved the selection of a random sample of 40 addresses from within each PSU resulting in an initial sample of 25,600 addresses. The third stage involved the recruitment of all members of the household aged 50 years and over. Consequently, the response rate was defined as the proportion of households including an eligible participant from whom an interview was successfully obtained. A response rate of 62% was achieved at the household level. There were three components to the survey. Respondents completed a computer-assisted personal interview and a separate self-completion paper and pencil module which collected information that was considered sensitive. All participants were invited to undergo an independent health assessment at one of two national centres using trained nursing staff. Blood samples were taken during the clinical assessment with the consent of participants. A more detailed exposition of study design, sample selection and protocol are available elsewhere [19]. The present study sample included 500 healthy individuals: 125 for each of the four socio-economic classes: stable professional, any downward mobility, any upward mobility, and stable unskilled. Buffy coat or peripheral blood mononuclear cells (PBMC) samples were available for all the individuals. Overall, after DNA methylation data quality controls and sample filtering, 490 subjects were analysed in this study.

For the microarray, DNA samples were extracted from buffy coats using the QIAGEN GENTRA AUTOPURE LS (Qiagen, Crawley, UK). Bisulphite conversion of 500 ng of each sample was performed using the EZ DNA Methylation-Lightning™ Kit according to the manufacturer’s protocol (Zymo Research, Orange, CA). Then, bisulphite-converted DNA was used for hybridisation on the Infinium HumanMethylation 850 k BeadChip, following the Illumina Infinium HD Methylation protocol. Briefly, a whole-genome amplification step was followed by enzymatic end-point fragmentation and hybridisation to HumanMethylation EPIC Chip at 48 °C for 17 h, followed by single nucleotide extension. The incorporated nucleotides were labelled with biotin (ddCTP and ddGTP) and 2,4-dinitrophenol (DNP) (ddATP and ddTTP). After the extension step and staining, the BeadChip was washed and scanned using the Illumina HiScan SQ scanner. The intensities of the images were extracted using the GenomeStudio (v.2011.1) Methylation module (1.9.0) software, which normalises within-sample data using different internal controls that are present on the HumanMethylation 850 k BeadChip and internal background probes. The methylation score for each CpG was represented as a β value according to the fluorescent intensity ratio representing any value between 0 (unmethylated) and 1 (completely methylated).

#### Understanding society

The study sample consisted of participants from the United Kingdom Household Panel Study (UKHLS), also known as Understanding Society [57], an ongoing longitudinal, nationally representative study of the UK, designed as a two-stage stratified random sample of the general population. While Understanding Society is a panel survey, the data used here consist of two pooled cross-sectional waves where a nurse collected blood samples from the respondents, among other physiological measures. The eligibility criteria for collecting blood samples were: (a) participation in the previous main interviews in England (had participated in all annual interviews between 1999 (BHPS wave 9) and 2011–2013 (Understanding Society wave 2 and 3); (b) age 16 and over; (c) living in England, Wales, or Scotland. From the potential pool of 6337 survey respondents, eligibility requirements for epigenetic analyses meant that the samples for DNA methylation measurement were restricted to participants of white ethnicity, resulting in 1175 subjects; more details can be found elsewhere [31]. Details about laboratory analyses for DNAm and how to access raw data can be found at the Understanding Society web site.

(https://www.understandingsociety.ac.uk/documentation/mainstage/dataset-documentation/variable/epigenetics).

For the GSE174818 (Covid-19 case–control) study, details of sample characteristics and laboratory methods for DNAm and biomarker analyses are described in the original publication [32].

The Health and Retirement Study (HRS) is a nationally representative longitudinal survey of more than 37,000 individuals U.S.A. [35]. The survey has been fielded every 2 years since 1992 and was established to provide a national resource for data on the changing health and economic circumstances associated with ageing at both individual and population levels. The cohort study is focussed on four broad topics: income and wealth; health, cognition, and use of healthcare services; work and retirement; and family connections. HRS data are also linked at the individual level to administrative records from Social Security and Medicare, Veteran’s Administration, the National Death Index, and employer-provided pension plan information. Since 2006, data collection has expanded to include genetic and epigenetic biomarkers. DNA methylation assays were done on a non-random sub-sample (*N* = 4018) of people who participated in the Health and Retirement 2016 Venous Blood Study. In this study, we used the sub-sample in which health assessment at the follow-up was available (*N* = 2146). The sample is 60% female and has a median age of 67 years and ranges in age from 50 to 100. It has racial diversity: non-Hispanic White and others (81.1%), non-Hispanic Black (10.0%), and Hispanic (8.9%). The sample is weighted to be representative of the U.S. population. DNA methylation data are based on assays using the Infinium Methylation EPIC BeadChip completed at the Advanced Research and Diagnostics Laboratory at the University of Minnesota. Samples were randomised across plates by key demographic variables (age, cohort, sex, education, race/ethnicity) with 39 pairs of blinded duplicates. Analysis of duplicate samples showed a correlation > 0.97 for all CpG sites. The minfi package in R software was used for data pre-processing, and quality control; 3.4% of the methylation probes (*n* = 29,431 out of 866,091) were removed from the final data set due to suboptimal performance (using a detection p value threshold of 0.01). Analysis for detection p value failed samples was done after removal of detection p value failed probes. Using a 5% cut-off we removed 58 samples. We also removed sex-mismatched samples and any controls (cell lines, blinded duplicates).

The Northern Ireland Cohort for the Longitudinal Study of Ageing (NICOLA) is a longitudinal cohort representative of the non-institutionalised population of Northern Ireland aged 50 years and older (*N* = 8504) [34]. The study, which was established in 2013, has three main components: a computer aided personal interview (CAPI), a self-completion questionnaire and health assessment. Dietary intake was also assessed by a food frequency questionnaire. The CAPI was extensive in scope and included assessment of demographic, social and health-related factors. Measures of cardiovascular, physical, cognitive, and visual function were determined, and a biobank of biological samples collected. DNA samples were extracted from buffy coats by Eurofins Scientific and normalised using PicoGreen quantitation. Bisulphite conversion of 500 ng of each sample was performed using the EX Zymo Methylation Kit (Zymo Research, Orange, CA) using the alternative overnight incubation conditions provided in the published protocol for use with the Illumina Infinium MethylationEPIC kit (Illumina USA). Then, bisulphite-converted DNA was used for hybridisation on the Infinium MethylationEPIC BeadChip array (Illumina, USA) following the manufacturer’s instructions, with arrays run on an Illumina HiScan. The intensities of the images were background adjusted and extracted as beta values using the GenomeStudio (v.2011.1) Methylation module (1.9.0) software.

### DNA methylation data pre-processing and quality controls

For all the studies but HRS (pre-processing procedure described previously), raw DNAm data were pre-processed and normalised using in-house software written for the R statistical computing environment, including background and colour bias correction, quantile normalisation, and BMIQ procedure to remove type I/type II probes bias, as described elsewhere [58]. DNAm levels were expressed as the ratio of the intensities of methylated cytosines over the total intensities (β values). Samples were excluded if the bisulphite conversion control fluorescence intensity was less than 10,000 for both type I and type II probes. Methylation measures were set to missing if the detection *P* value was greater than 0.01. Additionally, the set of cross-reactive and/or polymorphic (with minor allele frequency greater than 0.01 in Europeans) CpGs (*N* = 39,238) described by Chen et al. [59] was excluded due to the low reliability of methylation measure.

The Fernández-Sanlés methylation risk score (MRS) was computed as a standardised weighted sum of 34 CpG sites, with weights defined by the estimates described by the authors in the Supplementary material of their original publication [1]. DNAmGrimAge and other epigenetic clocks were computed using Steve Horvath online DNAmAge calculator.

(https://horvath.genetics.ucla.edu/html/dnamage/).

### Outcome definition

In EPIC Italy and EXPOsOMICS, incident CVD cases were identified from hospital discharge databases when the clinical record reported the International Classification of Diseases (ICD), Ninth Revision, Clinical Modification code 410, or ICD 410 plus the procedure codes for coronary revascularisation (e.g. percutaneous trans-luminal coronary angioplasty and coronary artery bypass surgery), including. Suspect CHD events were confirmed when myocardial infarction (MI), acute coronary syndrome, ischaemic cardiomyopathy, coronary or carotid revascularisation, and ischaemic or haemorrhagic stroke were reported in the records, supported by information on onset symptoms, levels of cardiac enzymes and troponins, and electrocardiographic data coded according to the Minnesota Code. Cases were cross-checked with mortality files to identify fatal and nonfatal cases (the latter defined as alive 28 days after diagnosis). Study participants with CHD at cohort entry were identified from the baseline questionnaire, from linkage with hospital discharge records, or by direct examination of clinical records, and were excluded from this study. In NICOLA and HRS, we defined CVD events accordingly with the definition used in the training set (EPIC Italy).

### Statistical analyses

#### Development and validation of DNAm surrogates

We developed DNAm surrogates for BMI, systolic and diastolic blood pressure, and ten blood measured biomarkers. We used the EPIC Italy data set randomly split into training (*N* = 1352; 75% of the sample) and test set (*N* = 451; 25% of the sample). For each risk factor/biomarker, we created a DNAm surrogate through a three-step procedure:

1. We identified risk factors/biomarkers showing significant differences across EPIC Italy centres (Turin, Varese, Naples, Ragusa) via ANOVA analyses. We employed a linear model with a random intercept component, accounting for differences across centres for this subset of biomarkers, consisting of all but PAI-1, CRP, D-dimer, and triglycerides. We used a fixed-effect linear model for the other biomarkers.
2. Log-transformed risk factors/biomarkers were regressed on DNAm through a linear model adjusted for age, gender (fixed effect), and centre of recruitment (random effect, where necessary) to identify the top 1% ranked CpGs based on the *P* value.
3. DNAm surrogates of risk factors/biomarkers were constructed, regressing the response variables on the top 1% CpG sites, adjusting for sex and age. Finally, we applied L1 penalised estimation for enforcing sparsity in the regression coefficients employing the LASSO procedure [60] or the corresponding penalised mixed model [61] (for the biomarkers showing difference by centre) depending on the biomarker. For the latter method, ad hoc R routines were devised: the source code is freely available in the form of an R package at https://github.com/AndreaCappozzo/mixedelnet.

We validated the DNAm surrogates investigating their association (Pearson correlation coefficients) with the corresponding measured risk factor/biomarker in the EPIC Italy testing set (*N* = 451, 25% of the sample), and four additional independent studies: Understanding Society (*N* = 1174), TILDA (*N* = 490), EXPOsOMICS CVD (*N* = 315), and GSE174818 (*N* = 128). We used fixed-effect meta-analysis (inverse variance weights) to combine the results across the four validation data sets into a single estimate. As a result, we defined as ‘validated’ DNAm surrogates with significant associations (*P* < 0.05) in both EPIC Italy and the combined validation sets. As further validation, we investigated the correlation of our newly developed DNAm surrogates with those previously developed for BMI, HDL cholesterol [23], and PAI-1 [11].

##### Derivation of DNAmCVDscore

We developed a blood DNAm-based biomarker (that integrates several DNAm surrogates) for predicting the risk of future CVD events named *DNAmCVDscore*. We used a Cox regression model with elastic net regularisation to regress the time from recruitment to CVD event, and for selecting the most critical features from 60 (standardised: mean = 0, sd = 1) previously described blood DNAm surrogates.

The best λ parameter was derived from tenfold cross-validation to minimise the Harrell’s concordance C-index. The overall procedure includes 1,000 permutations using 80% of the whole EPIC Italy data set each time (*n* = 1443). The DNAm surrogates comprising the *DNAmCVDscore* were selected among those with nonzero coefficients in at least half of the permutations. Finally, *DNAmCVDscore* was computed as a linear combination of the selected DNAm surrogates where weights correspond to the average (nonzero) coefficient among the 1,000 permutations.

##### Validation of DNAmCVDscore and comparison with MRS, SCORE2 and DNAmGrimAge

We validated the *DNAmCVDscore* in three independent data sets: EXPOsOMICS CVD, NICOLA, and HRS. Since the EXPOsOMICS CVD set is designed as a case–control study nested in a cohort, we ran logistic regression analyses, and we evaluated the predictive performance of *DNAmCVDscore* through ROC curve analysis. Contrarily, in NICOLA and HRS (cohort studies) we run Cox proportional hazard regression models and we evaluated the prediction performance through the Harrell’s C-index.

We compared the performance of five models:

1. Based on *DNAmCVDscore* (adjusted for matching parameters in EXPOsOMICS CVD).
2. Based on MRS (adjusted for matching parameters in EXPOsOMICS CVD).
3. The SCORE2 prediction model based on chronological age, sex, diabetes, smoking, systolic blood pressure, total and HDL cholesterol, adjusting for matching parameters.
4. An enriched version of SCORE2, denoted with SCORE2 + *DNAmCVDscore*, in which *DNAmCVDscore* is included in the set of covariates.
5. Based on DNAmGrimAge (adjusted for matching parameters in EXPOsOMICS CVD).

In EXPOsOMICS CVD, to investigate the predictive performance of the five composite biomarkers at different time points, we computed the area under the ROC curve (AUC), sensitivity, and specificity as a function of the time from recruitment to diagnosis, right-censoring follow-up at constant intervals of one year from 18 to 2 years. Confidence intervals for AUC were computed according to De Long et al. [62].

##### DNAm surrogates and DNAmCVDscore versus COVID-19 case–control status and severity

As an additional sensitivity analysis, despite being out of the main scope of this work, we investigated the usefulness of using DNAm surrogate biomarkers in epidemiological studies on COVID-19 using the GSE174818 data set (101 patients with COVID-19 infection and 26 controls hospitalised with respiratory problems). Specifically, we investigated the association of BMI and blood measured CRP with COVID-19 case–control status and severity (using the GRAM score as a proxy), and we compared the results with those obtained using their DNAm surrogates (DNAmBMI and DNAmCRP). Finally, since CVDs and COVID-19 share several risk factors [50] we investigated the association of the *DNAmCVDscore* with COVID-19 case–control status and severity. We used logistic and linear regression models adjusted for age and gender to investigate the association with case–control status and GRAM score, respectively.

## Supplementary Information

**Additional file 1**. Supplementary Results and Supplementary Figures S1, S2, S3, and S4.

**Additional file 2**. Supplementary Tables S1, S2, and S3.

## Data Availability

DNAm data used in this study are available at the GEO repository under accession numbers GSE51032 (subset of EPIC Italy) and GSE174818 (COVID-19 case–control). DNAm data from TILDA, EXPOsOMICS, NICOLA, and EPIC Italy are available upon request at the study PIs/ Data Access Committees. For HRS, three categories of data: public, sensitive, and restricted, can be accessed through procedures described on the HRS website (hrsonline.isr.umich.edu). Understanding Society data were collected by NatCen, and the genome-wide scan data were analysed by the Wellcome Trust Sanger Institute. Information on how to access the data can be found on the Understanding Society website https://www.understandingsociety.ac.uk/.

## Abbreviations

BMI: Body mass index
CRP: C-reactive protein
CVD: Cardiovascular disease
DNAm: DNA methylation
EPIC: European Prospective Investigation into Cancer and Nutrition
EWAS: Epigenome-wide association studies
GDF15: Growth differentiation factor 15
HGF: Hepatocyte growth factor
HRS: The Health and Retirement Study
MRS: Methylation risk score
NICOLA: Northern Ireland Cohort for the Longitudinal Study of Ageing
NCDs: Non-communicable diseases
PAI-1: Plasminogen activator inhibitor-1
sd: Standard deviation
SKR3: Serine/threonine-protein kinase receptor 3
UKHLS: United Kingdom Household Panel Study
WBC: White blood cells
