# Supplementary material for: A blood DNA methylation biomarker for predicting short-term risk of cardiovascular events
Source: Clin Epigenetics. 2022 Sep 29;14:121. doi: 10.1186/s13148-022-01341-4 (PMC9521011; doi:10.1186/s13148-022-01341-4)

**Supplementary material for A blood DNA methylation biomarker for predicting short-term risk of cardiovascular events**

Andrea Cappozzo^1^, Cathal McCrory^2^, Oliver Robinson^3^, Anna Freni Sterrantino^3,4^, Carlotta Sacerdote^5^, Vittorio Krogh^6^, Salvatore Panico^7^, Rosario Tumino^8^, Licia Iacoviello^9,10^, Fulvio Ricceri^11,12^, Sabina Sieri^6^, Paolo Chiodini^13^, Gareth J McKay^14^, [Amy Jayne McKnight](https://pubmed.ncbi.nlm.nih.gov/?term=McKnight+AJ&cauthor_id=31009935)^14^, Frank Kee^14^, Ian S Young^14^, Bernadette McGuinness^14^, Eileen M Crimmins^15^, Thalida Em Arpawong^15^, Rose Anne Kenny^2^, Aisling O’Halloran^2^, Silvia Polidoro^16^, Giuliana Solinas^17^, Paolo Vineis^3^, Francesca Ieva^1,18^, Giovanni Fiorito^2,3,17, *^

**Supplementary Results**

*Sensitivity leave-one-out analysis*. We performed a sensitivity analysis to evaluate whether one of the ten DNAm surrogate biomarkers comprising the *DNAmCVDscore* drives the results described in the previous section. First, the *DNAmCVDscore* was re-computed ten times, excluding one DNAm surrogate each time. Then, AUC and 95% CI were calculated at different time points right censoring follow-up length as described previously. The results presented in **Table S1** show that the AUC obtained using ten DNAm surrogates is generally higher than those obtained excluding one of them. However, none of the biomarkers significantly reduces the AUC when excluded (according to the DeLong test), suggesting that all the ten DNAm surrogates contribute to predicting CVD events.

*Sensitivity analysis on DNAmHDL*. Since DNAmHDL has the higher weight among the 10 DNAm surrogates composing the *DNAmCVDscore* (**Table 3**), we have evaluated its prediction performance alone. Also, we computed the prediction performance of SCORE2 + DNAmGrimAge and SCORE2 + DNAmCVDscore + DNAmGrimAge comparing them with SCORE2 + *DNAmCVDscore*. The results presented in **Table S2** show that the AUC obtained using DNAmHDL alone is lower than that obtained *DNAmCVDscore* and that there was not a significant increment in prediction using SCORE2 + *DNAmCVDscore* + DNAmGrimAge compared with SCORE2 + *DNAmCVDscore*.

*Correlation of DNAmCVDscore with epigenetic clocks*. We computed the Pearson correlation coefficients (meta-analysis of the five studies) between *DNAmCVDscore* and previously developed epigenetic clocks. Although *DNAmCVDscore* was not explicitly trained on chronological age, it is highly correlated with age (R = 0.41) and four epigenetic clocks (R range from 0.35 to 0.56), DNAmGrimAge being the one with the highest correlation. In **Figure S3,** we present the correlation heatmap.

*DNAm surrogates and DNAmCVDscore vs COVID-19 case-control status and severity*. In the GSE174818 dataset, BMI was not significantly different when comparing COVID-19 cases with matched controls, whereas the DNAm surrogate for BMI was significantly associated with COVID-19 case-control status: OR per one standard deviation increase = 2.64 (95% CI 1.56; 4.77, *P* = 0.0006, **Table S3**). Among COVID-19 cases, DNAmCRP outperforms blood-measured CRP in predicting disease severity (GRAM score). The increase in the GRAM score were 17.1 (8.6; 25.6, *P* = 0.0002, **Table S3**) and 9.9 (1.1; 18.7, *P* = 0.03, **Table S3**) for DNAmCRP and blood measured CRP, respectively. Finally, *DNAmCVDscore* was (borderline significantly) associated with COVID-19 case-control status: OR per one standard deviation increase = 1.84 (95% CI 0.96; 3.65, *P* = 0.07, **Table S3** and **Figure S2a**), and GRAM score severity index. The estimate from the linear regression (interpretable as an increase in the GRAM score for one standard deviation increase in *DNAmCVDscore*) was 16.35 (95% CI 1.36; 31.04, *P* = 0.03, **Table S3** and **Figure S4b**).

**Figure S1**: Scatterplots of observed (y-axis) *vs* predicted (x-axis) standardized values for the nine DNAm surrogates validated within this study. Points color indicate the validation dataset; red: TILDA, blue: Understanding Society, green: GSE174818, purple: EXPOsOMICS CVD. Dotted lines indicate dataset specific least squared regression lines.


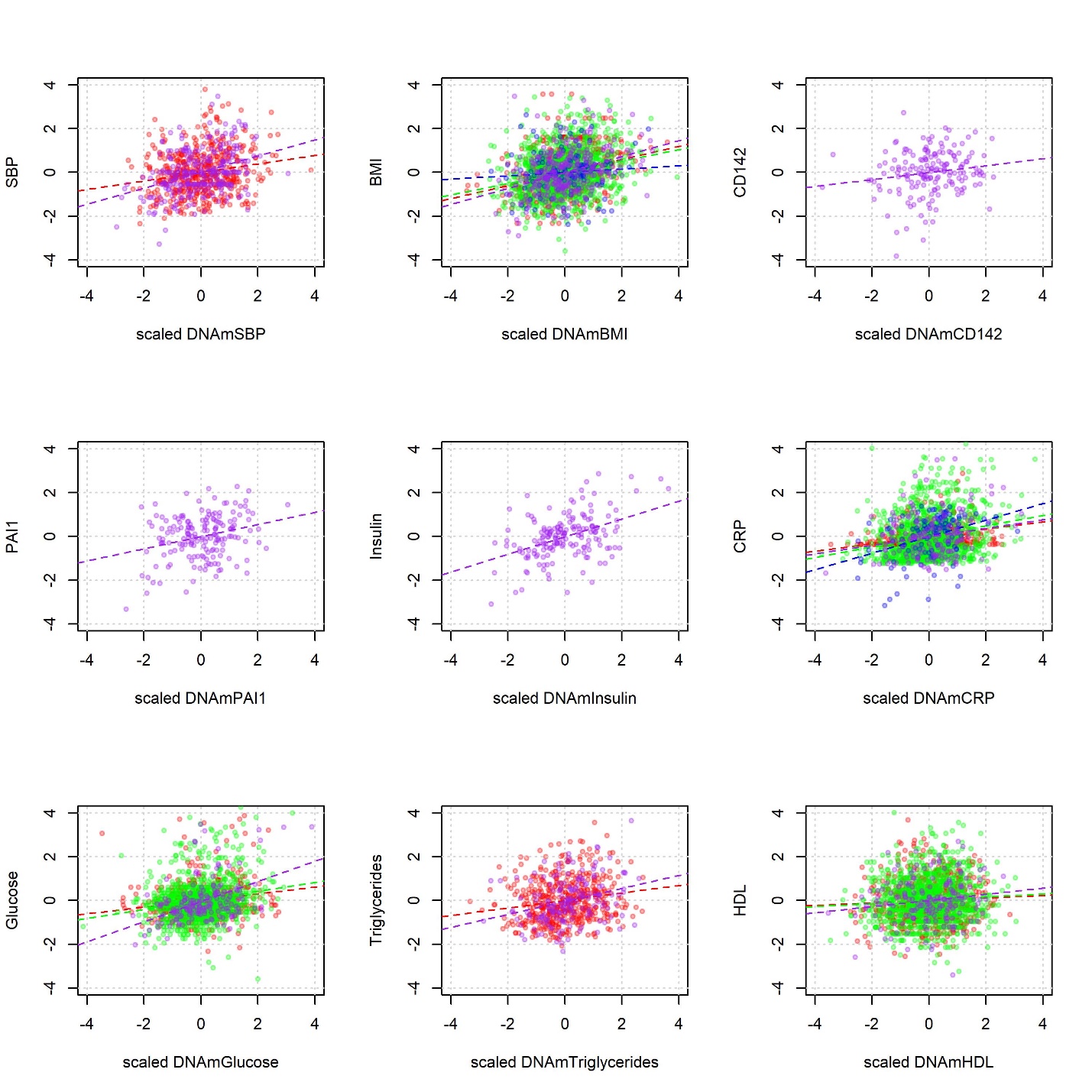


**Figure S2**: Correlation heatmap with mutual Pearson correlation coefficients among *DNAmCVDscore* and the 10 DNAm surrogates composing it.


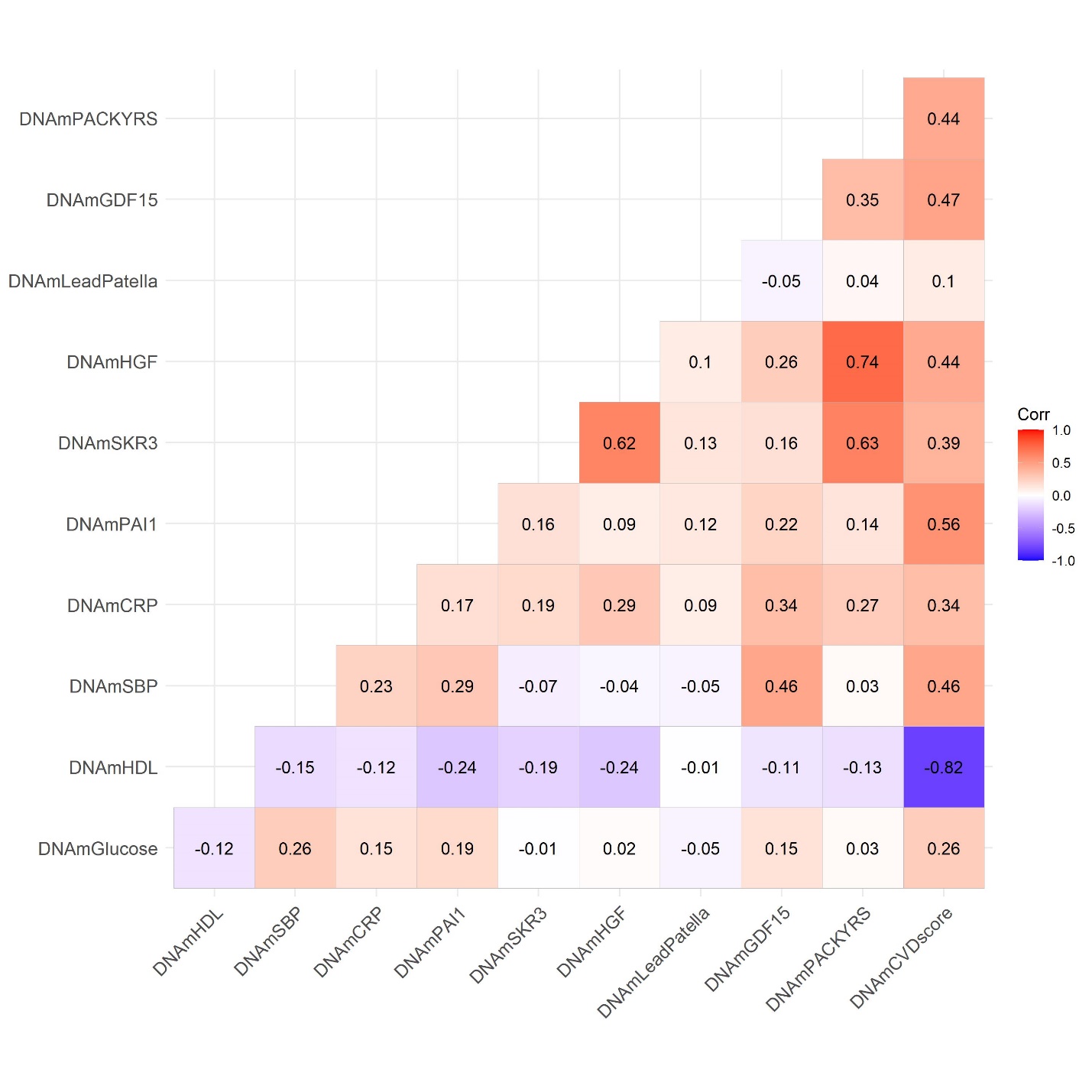


**Figure S3**: Correlation heatmap with mutual Pearson correlation coefficients among *DNAmCVDscore* and epigenetic clocks


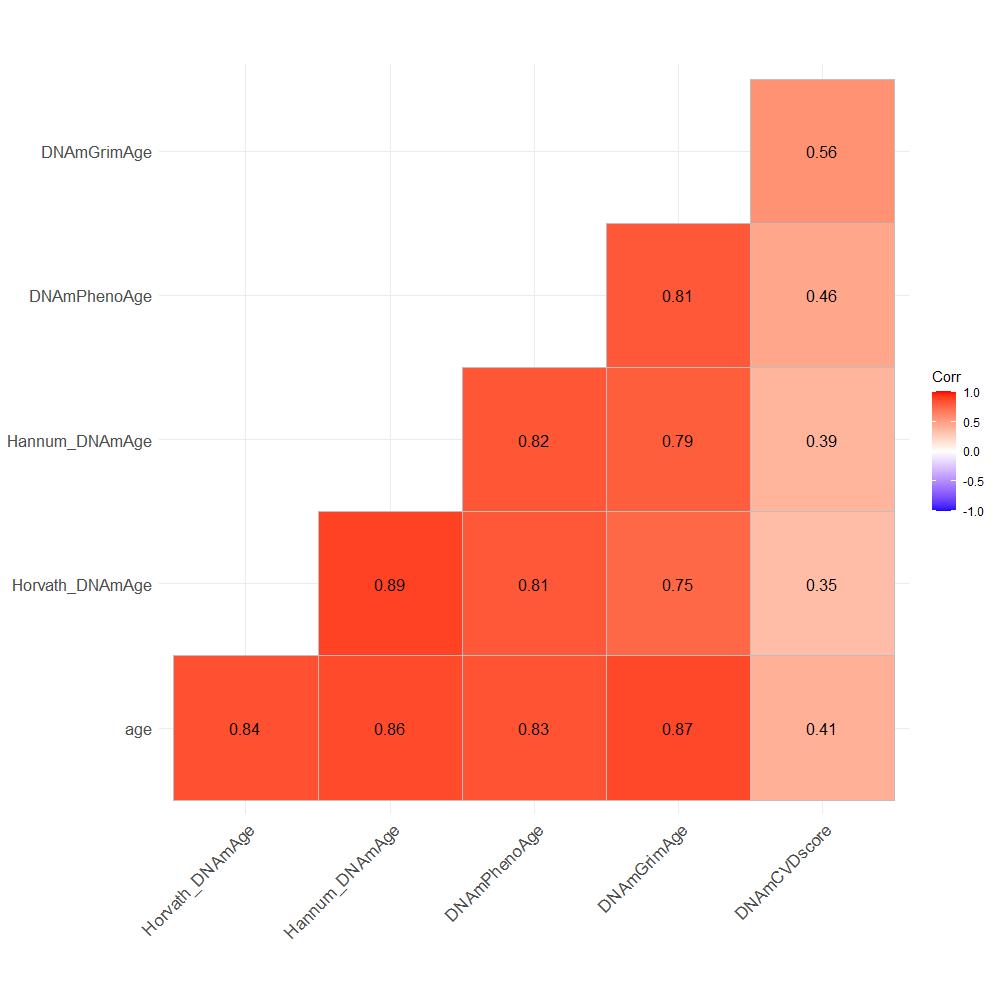


**Figure S4**: a) Violin plot of standardised *DNAmCVDscore* vs COVID-19 case-control status; b) scatterplot and regression line for *DNAmCVDscore* vs COVID-19 severity (GRAM score).


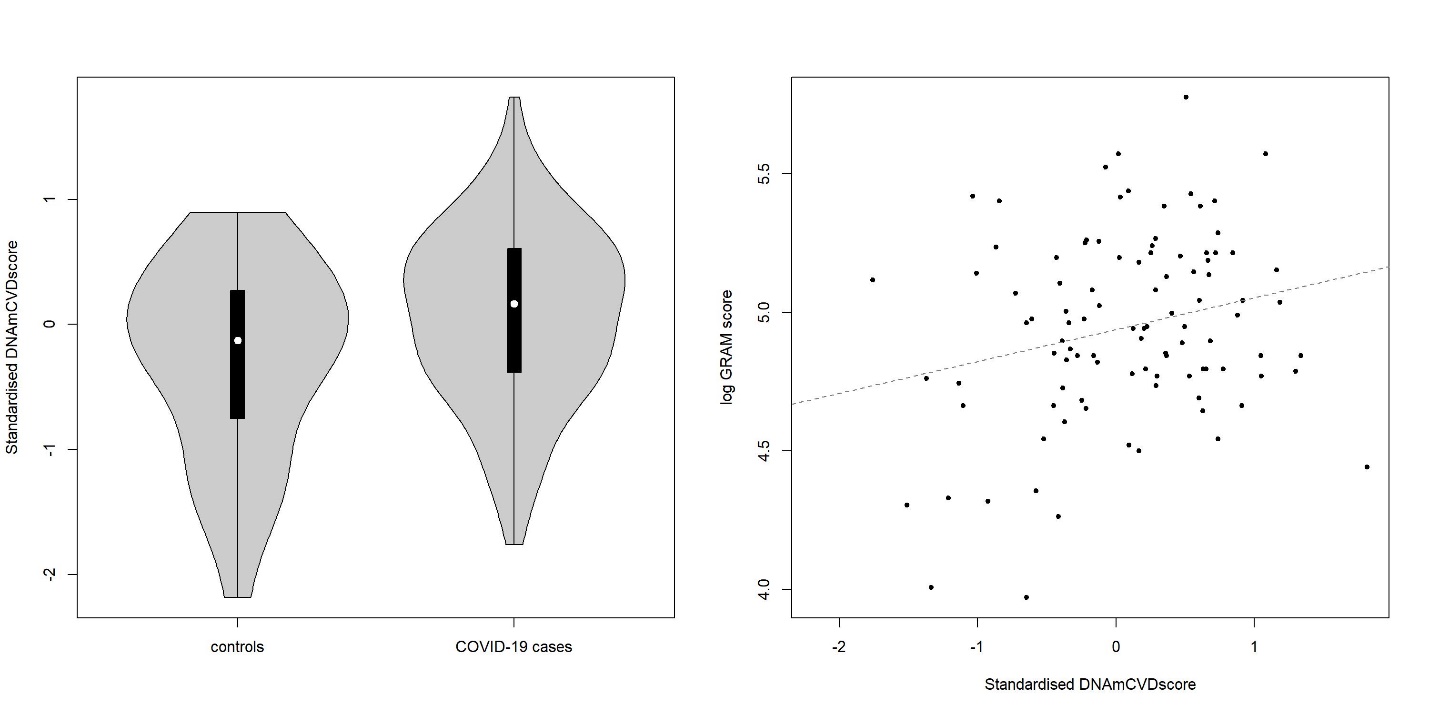

Supplement: Supplementary file 1 — Additional file 1. Supplementary Results and Supplementary Figures S1, S2, S3, and S4. [file 13148_2022_1341_MOESM1_ESM.docx]
